# Understanding weight regain: Insights from Saudi patients on factors influencing post-metabolic and bariatric surgery outcomes: A qualitative study

**DOI:** 10.1371/journal.pone.0340120

**Published:** 2026-02-12

**Authors:** Suhair Al-Ghabeesh, Ahmad Rayan, Enas A. Assaf, Mirna Fawaz, Abbass AlBay, Hanan Alyami

**Affiliations:** 1 Faculty of Nursing, Al-Zaytoonah University of Jordan, Amman, Jordan; 2 Faculty of Nursing, Zarqa University, Zarqa, Jordan; 3 Nursing College, King Khalid University Khamis Mushait, Abha, Saudi Arabia; 4 Faculty of Health Sciences, Nursing Department, Beirut Arab University, Beirut, Lebanon; 5 College of Nursing, Department of Medical and Surgical Nursing, Princess Nourah Bint Abdulrahman University, Riyadh, Saudi Arabia; Shahid Beheshti University of Medical Sciences School of Medicine, IRAN, ISLAMIC REPUBLIC OF

## Abstract

**Background:**

Weight restoration is still possible even if Metabolic and Bariatric Surgery is the most efficient therapy for extreme obesity. While the non-controllable causes of weight regain have been investigated, the controllable causes of weight regain have received less attention.

**Purpose:**

The aim of this study was to explore the factors contributing to weight regain after Metabolic and Bariatric Surgery as perceived by patients.

**Methods:**

A qualitative descriptive research design used, where semi-structured interviews were carried out.

**Results:**

The thematic analysis gave rise to five themes. The first theme was titled “Economic challenges,” and it highlighted the challenges experienced by the patients in maintaining a healthy diet. The second theme, “Emotional instability and occupational stress,” reflects the psychological concerns that patient’s experience, which hinder them from keeping a healthy lifestyle. The “Social influence” theme focused on the environment of the patients, which enhanced cravings, and the “Lack of support” theme highlighted the negative perspective of the social network of the participants towards the patients’ surgery and desired lifestyle. Finally, the “False beliefs and decreased physical activity” theme focused on how the perception of the patients towards the results or effects of the surgery led to reduced physical activity, which they perceived as leading to weight gain.

**Conclusion:**

Despite being successful, weight regain can still happen following bariatric surgery. In order to promote long-term weight loss sustainability, the results of this paper reinforce the idea that persons who have had Metabolic and Bariatric Surgery ought to be educated of the controllable variables linked with weight rebound, future research address the long term effect and the weight control factors following the surgery are highly recommended.

## Introduction

The World Health Organization [1] estimates that by 2025, around 167 million individuals, both adults and children, will experience declining health due to being overweight or obese. The highest rates have been reported in the Middle East and North Africa; for instance, in Saudi Arabia, about 70% of both genders are classified as overweight, and 35% are considered obese [1]. According to the survey executed by Al-Enazi and Al-Falah [2], Metabolic and Bariatric Surgery (MBS) is gaining popularity in Saudi Arabia. MBSis more effective than dietary changes for maintaining long-term weight reduction and enhancing general wellbeing and quality of life (QoL). The amount of weight lost following MBS differs from person to person. After reaching their minimum weight, the majority of patients tend to gain some weight [3–6]. According to previous research, the occurrence of weight regain in the bariatric population varies greatly. While other research findings, documented that 20–24% of patient populations have acquired back more than 15% of their body mass 5 years after gastric bypass or sleeve gastrectomy [7]; the Longitudinal Assessment of Metabolic and Bariatric Surgery research (LABS) demonstrates an average weight regain of about 4% of their body mass 3–7 years after surgery [8]. Whereas a distinction for measuring weight regain and identification of prevalence of re-occurrence is difficult to estimate [9,10]. Additionally, around 20% to 35% of patients encounter weight regain, influenced by factors such as surgical approaches and the time frame after surgery. Analysis following the nadir point demonstrated that this weight regain substantially affects the worsening of related health conditions, including hypertension and dyslipidemia when the greatest weight loss surpasses 20%, and diabetes when it exceeds 25% [11]. Furthermore, several elements contribute to post-operative weight outcomes and appetite control including heredities, a boost in gut hormones, i.e., glucagon-like-peptide-1 (GLP-1) and peptide YY (PYY), as well as a reduction in ghrelin secretion. Lost weight may be recovered due to post-bariatric hypoglycemia or anatomical failure associated with surgical operations like gastro-gastric fistula or dilated gastric pouch. Moreover, an inadequate diet, mental instability, hyperplasia, substance use, sedentary life and poor nutritional maintenance could stimulate weight regain after MBS [12]. Additionally, weight regain has been associated with several factors, including preoperative Body Mass Index (BMI). Heavier individuals tend to exhibit lower percentages of initial and excess weight loss, regardless of whether their BMI is above or below 60 kg/m^2^. This effect was particularly noticeable after the initial 12-month rapid weight loss phase, during which less obese individuals (BMI < 50) continued to lose weight, while those with a higher BMI (BMI ≥ 50) experienced significant weight regain [13]. In addition to gender, advanced age, existing co-morbidities, as well as consistency and intensity of post-surgery follow-up [14–16]. Numerous studies have proposed medical, socio-demographic, and psychosocial factors to effect weight loss after MBS. Furthermore, the type of operation and procedure selected must be personalized for each patient based on their unique circumstances, which include, but are not limited to, BMI, medical considerations, and the presence of comorbidities [9,11,17].

A qualitative study shows that after surgery, patients either enjoy long-standing benefits of their surgery even after 10 years, or face difficulties in controlling their weight. Some patients dread regaining weight and usually view it as an individual setback [18]. Weight regain has proven to be a complex phenomenon. In order to provide adequate support to these patients, it is essential to understand the reasons and mechanisms behind weight regain, as well as the challenges associated with weight management after MBS. Therefore, conducting a qualitative research on patients’ insights may help identify the barriers and facilitators involved in this process. Consequently, this study aims to address the following question: “What factors do patients perceive as influencing weight regain after MBS?”

## Methods

### Study design

A qualitative descriptive research design based on Husserl’s descriptive phenomenology in order to get insight into the patients’ motivations, lived experiences and behaviours that have contributed to weight regain post-surgery [19]. Husserl’s descriptive phenomenology method is a qualitative approach to studying the lived experiences by suspending researcher assumptions to describe the essential features of a phenomenon as it appears in consciousness. This method aims for a direct, unbiased account of how individuals experience a phenomenon, often using in-depth interviews to gather rich, detailed descriptions. Two experienced researchers in qualitative research conducted in-depth, individualized semi-structured interviews to explore perceptions, feelings, and opinions about postoperative weight regain was chosen. The inductive approach enabled the generation of new insights and understanding from the material [20].

### Setting and population and sampling

Throughout October first 2022 and end of December 2022, treatment-seeking patients at the obesity clinic in Riyadh, Saudi Arabia, were purposively sampled to find respondents. The primary care doctor or an obesity specialist had recommended each individual for medical obesity therapy. Eligible individuals were personally invited to enroll in the research by the hospital personnel at the obesity center. A body mass index (BMI) of 35 kg/m2 or above, the age of 18, one year from the time of operation, and a weight restoration following MBS of at least 10% were requirements for eligibility. Printed and spoken study details were presented to obtain participants’ consent to voluntary participate in the study which adhered to the Declaration of Helsinki. Four potential participants chose not to participate due to time constraints. Ultimately, a total of 18 participants were interviewed, as saturation was reached.

### Demographic characteristics

A demographic questionnaire including the participant’s age, gender, employment, educational level, and marital status was used.

### Semi-structured interviews

Patients who were willing to participate were invited to take part in semi-structured interviews after completing the demographic data form. These interviews were conducted by two researchers (who are co-authors in this manuscript), to gather qualitative data about the patients’ perspectives of the causes of weight regain. The setting for the conversations was chosen with care to ensure the comfort and confidentiality of the participants. The session had a flexible schedule and had been worked out in advance. The sessions were scheduled at convenient times and days for the patients to avoid any disruptions or anxiety. During the session, the researchers presented the study and went through the meeting’s purpose. The participants were informed that this talk was entirely private and that any texts that were made public would not contain any identifying information about the participants and would instead be encoded for confidentiality which adhered to the Declaration of Helsinki. The participants were made aware that they might discontinue the study at any time. The participants were informed that only the investigators would be permitted to listen to the live recordings and that they would be kept safe, however the talks were recorded after permission. The interviews were conducted and transcribed in Arabic, which is the official language of the country. Later the transcripts were translated and back-translated by two members of the research team who are bilingual. To ensure that adequate information was gathered, the interviews were each timed to go no more than 30 minutes In addition to review the answers with each participants to verify the correct messages. While in the coding process for the interviews, each of the two researchers (who conducted the interviews) independently coded the interviews and recorded their codes separately. They then met to discuss their interpretations and the emerging themes. The two researchers agreed on with 95% of the overall codes and themes. Furthermore, theoretical saturation was considered throughout the processes of data collection and analysis.

#### Interview guide.

1. How much weight did you lose one year after MBS?
2. How do you describe your life style after MBS?
3. How much do you follow a regular less fat diet after one year of the MBS?
4. How do you describe your dietary habits after one year of the MBS?
5. Do you suffer from any mental health disorder (anxiety, stress, depression) after one year of the MBS?
6. What are the barriers to lose weight after MBS? (Social factors, (e.g., eating practices with colleagues), personal characteristics (e.g., self-efficacy, motivation, knowledge) and features of the physical environment (e.g., lack of availability of healthy food in onsite cafeterias, vending machines), eating behaviours.
7. What type of food do you eat after MBS?”
8. Healthy eating behaviours were defined in line with guidelines. They encompass: (i) the timing and frequency of eating; (ii) meal composition; (iii) food composition; (iv) the habitual average intake of energy and essential non-energy yielding nutrients. Are the four components present in your life-style?

However, researchers were open to what participants wanted to share. Study guide is available in the supporting information to this manuscript.

#### Data analysis and trustworthiness.

Thematic analysis was used to create themes that represented the patients’ points of view in order to address the study objectives. Concurrent analysis was done to make sure that, in light of these findings; the ideas produced by the theme analysis were accurate and pertinent. At about the same time of day and in a similar location, the researcher spoke with each participant in an informed and approachable manner. Additionally, the identical set of questions was asked to each participant, and the interviewer addressed all pertinent topics without hiding any interview-related information.

### Ethical approval

The academic institutional review board of Rayak University Hospital Saudia Arabia, IRB no. ECO-R-360 approved the study. The submitted documents included details about the study’s objectives, procedures, techniques, participant involvement, ethical considerations, methods for ensuring investigator well-being, and risk assessment within the hospital context, which adhered to the Declaration of Helsinki. Once the project received final approval from the clinic, it proceeded. Additionally, the obesity clinic obtained informed consent from the study participants. All participants were required to sign a consent form before taking part in the study. Throughout the research process, the privacy, anonymity, and autonomy of each study participant were upheld

## Findings and results

The objective of this qualitative study was to identify relationships that contest, complement, and develop each other through a series of interviews. The thematic categorization maintains its validity since the specified categories are founded on a framework that is consistent with the viewpoints of the participants.

### Sociodemographic characteristics

The sample of this study was made up of 18 patients with a mean age of 32.78, who have undergone MBS, where, 6 (33.3%) of them were males, while 12 (66.7%) were females. 15 (83.3%) where the majority were university degree holder and employed (Table 1).

**Table 1:** Patient characteristics.

		N	%
**Gender**	Male	6	33.3
	Female	12	66.7
**Educational Level**	Primary School	2	11.1
	Secondary School	1	5.6
	University	15	83.3
**Employment**	Unemployed	5	27.8
	Employed	13	72.2
**Marital Status**	Single	4	22.2
	Married	13	72.2
	Divorced	1	5.6
**Age**	M + SD	95%CI	S.E
	32.78 ± 5.70	(29.94,35.61)	1.34

### Qualitative findings

The thematic analysis gave rise to five themes. The first theme was titled; “Economic challenges” and it highlighted the challenges experienced by the patients in maintaining a healthy diet. The second theme, “emotional instability and occupational stress” reflects the psychological concerns that patient’s experience, which hinders them from keeping a healthy lifestyle. The “social influence” theme focused on the environment of the patients, which enhanced cravings, and the “lack of support” theme highlighted the negative perspective of the social network of the participants towards the patients’ surgery and desired lifestyle. Finally, the “false beliefs and decreased physical activity” theme focused on how the perception of the patients towards the results or effects of the surgery led to reduced physical activity which they perceived as leading to weight gain.

#### Economic challenges.

The first theme that was prevalent from the process of thematic analysis reflected on how the global economic recession has affected the adherence of the patients to healthy diets and lifestyles. The participants have expressed how the inflation has hindered them from buying fresh produce and low-calorie products to satisfy their nutritional needs, and thus led them to revert to consuming cheaper, less healthy food options that have contributed to regaining weight. For instance, one of the patients said,

*“…you know after the whole recession started it became really hard for me to stick to a diet plan…it is really expensive to buy ingredients that you can both satisfy your cravings and stay healthy… it is way cheaper to access street food or make food at home which is usually saturated in fats…” (P3)*

Another patient also proclaimed,

*“…buying proteins is really expensive these days…I love eating dairy yet low calories or non-fat dairy is completely out of my financial abilities… so I buy regular dairy products which I think it is a huge reason why I regained weight… it is the same situation with buying oats or oat bread… regular bread is much cheaper… and umm… I end up consuming eat and it leads me to crave more sugar…” (P11)*

Another patient also had a similar experience,

*“…I have been living off street food because I can afford it more…it saves me more money to pay for rent and gas… cooking meals costs way more than buying fast food nowadays…that’s how I kind of regained the weight, I guess…” (P7).*

#### Emotional instability and occupational stress.

Another factor that the patients have highlighted which they perceive as a significant contributor to weight regain after MBS is mental health problems, which affected their eating behaviors. The majority of the patients in this study focused on their emotional states influenced their adherence to their diet plans and healthy lifestyles. For instance, one of the participants said,

*“…It is those times when I am not in a good place mentally when I feel I want to eat and eat and eat…I submit to my cravings whether it’s chocolate or pizza or whatever…It makes me feel better, I guess… though it is not healthy coping but I went through a lot after my surgery and I found my comfort in indulging, unfortunately… guess I should have found a better coping style…” (P12).*

Another patient also shared,

*“…I have suffered with depression for the past few years…usually when I am on a low, I stop eating, but this time it was different… I found myself craving food because I was deprived of my cravings for so long… it was the way that I was trying to make myself feel something… didn’t help it only added to the feelings of guilt and shame…” (P4).*

The patients in this study also associated their weight gain with the unhealthy eating habits that accompanies their work life. The patients recounted experiences relating to being overworked and stress and binge eating after their shift ends. For instance, one of the patients said,

*“…I work night shifts…I tend to be exhausted throughout the night…I find myself snacking through the shift to pass time or to regain some energy… when I get home I would not have the power to cook so I order some fast food and eat to keep my day going…” (P18).*

Another participant also proclaimed,

*“…my work is really fast paced and utterly stressful so I can’t eat all day…when I get home I eat a very large meal to satisfy the hunger that I would be going through all day and I end up overeating…” (P9).*

#### Social influence.

Moreover, the participating patients reflected on how their social context can influence their eating habits and overpower their will to adhere to their post-surgery diet plans. The participants emphasized the challenge of staying healthy within an environment where everyone is indulging in unhealthy eating habits. For instance, one of the patients said,

*“…it was really hard to look at your siblings and friends and family eating whatever they feel like eating…stuff that you really like eating and stay healthy…you can literally see your will breaking bit by bit… I could not resist..” (P1).*

Another patient said,

*“…the social gatherings and celebrations hit hard the most… whenever there is a birthday or a wedding or a holiday… everyone is happy enjoying food and I feel completely miserable for not being able to…it wasn’t long till I caved in and I felt the need to give myself the freedom to eat yet I did not feel good about it every time because I was noticing the weight gain slowly…” (16).*

A similar experience was also recounted,

*“…we live in a country where gastronomy is the center of the social life…Whenever I go out with my friends we are planning to eat or drink or do an activity around eating a couple meals or snacking…it wasn’t good for my diet…I’d either have to be antisocial or just give in to the lifestyle…” (P10).*

#### Lack of support.

Another factor that contributed to weight regain after MBS, according to the participating patients, was the lack of support from the direct family regarding the new lifestyle, and even not supporting the decision of the surgery in the first place. This has placed immense pressure on the participants to comply with dietary habits. For instance, one of the participants shared,

*“…my family does not understand what it takes for me to keep on track and to keep my mind and body balanced through all of this…when I decided to go for the surgery I was really struggling to convince my family how important this is for me and after that they won’t understand that they need to be involved in my journey to support me emotionally and actually through actions around the house…like give me options” (P14).*

Another patient also said,

*“…I mean my family has to kind of diet with me when I am around…like cooking stuff that can actually fit my diet… encourage me to stay healthy rather than just do whatever they want and when I ask them for help they’re like this is your choice not ours…” (P2).*

A similar experience was also shared by another patient,

*“…it is really hard to feel like you are on your own…you cave…my father especially is caught up in his ways and is not willing to bend some rules for my sake…so I am obliged to eat whatever they’re eating and it is fatty and full of carbs…when I ask for healthy food he would get to sarcastic and mock me… and I rely on them for food it’s not like I have any other choice…” (P17).*

#### False belief and decreased physical exercise.

The final theme that was prevalent from thematic analysis related to false beliefs about the effects of the surgery, which led to a decrease in engagement in physical activity and therefore, led to weight regain. The patients felt that they relied solely on the surgery to reduce their weight and gave up on the effort to exercise. For example, one of the patients said,

*“…I can’t believe I was so unrealistic in how I expected that the surgery alone would do the trick and I just felt like why would I have to exercise…instead I was just stick to my regular routine during the day and I guess that was not very good…” (P8).*

Another participant also said,

*“…the doctor told me that I would have to exercise…but I did not…when I first decided to go for the surgery I went in with the toxic optimism that it is the miraculous solution to my weight and that it is like a magic wand that will strip off all those kilos on its own…so I did not exercise and I ended up actually regaining all the weight that I would have potentially lost if I was more careful and keen…” (P15).*

## Discussion

This is the first qualitative research in Saudi Arabia to investigate the perspectives of these patients. Dietary patterns in Saudi Arabia have been moving towards a Western-style diet that is rich in fat, salt, and sugar, resulting in an increased obesity rate [21,22]. Social interaction in Saudi culture is based on food serving and food themes as gatherings, wedding celebrations revolve around food preparation and high-calorie food serving [21,22].

Five themes have resulted from the thematic analysis in this study, focusing on the lack of support, effect of emotional concerns, social influence, reduced physical activity, and economic challenges on weight regain after MBS.

The respondents had anticipated that having surgery would provide them long-term and control over their diet and weight. Patients anticipate that MBS would put an end to their battles with food and obesity. In this study, loss of control took the place of the early emotions of assurance and changes in dietary practices [23]. Respondents hypothesized that failure to follow dietary advice led to a decrease in managing their weight. Lack of dietary compliance, as seen by increased carbohydrate intake, higher fat consumption found in street food, and inferior nutritional quality, was found to be a major factor in weight gain after the recuperation from bariatric surgery. This was associated with economic challenges that the participants faced in obtaining more healthy and appropriate ingredients. Previous research has shown that variations in dietary compliance during the post-operative period may be linked to weight regaining in individuals who have undergone MBS [24]. The most obvious dietary factor linked to weight increase seems to be an increased carbohydrate intake. Although the origin of the carbs was not precisely characterized in all research, some have shown that a rise in the consumption of fluid calories and glucose from sources other than food were related to weight regain [25].

The participants in this study have reported episodes of binge eating due to stress and emotional distress after non-compliance. The feeling of “a twofold defeat”, originally being unable to sustain a healthy weight and then failing to attain a satisfactory post-operative outcome, exacerbated the psychological suffering that respondents felt after weight regaining. The guilt that individuals felt as a result of social weight stigma may have influenced ineffective coping mechanisms, including emotional eating, grazing, or limited eating, which are per research that links weight regain to unhealthy eating habits [26,27]. Instilled weight stigma and additional weight control challenges have been linked to poor self-esteem, unhealthy eating habits, drug use, and generalized poorer psychological adjustment. Even though bingeing or depressive symptoms appear to lessen following operation, our study found that issues with eating patterns and psychological health issues are the main causes of weight increase [28].

Due to the gastric constriction caused by bariatric surgery, an individual’s caloric intake increases through the ingestion of higher calorie-dense meals, fluids, or more meals that are regular. This illustrates that food patterns change gradually following bariatric operations instead of being constant, and some individuals might not be aware of the implications this will have on their long-term prognosis. In order to discover answers and solutions to the obstacles to sustaining compliance with dietary guidelines, individuals will likely need the continuing guidance and supervision of nutritionists and their bariatric team. Consequently, MBS by itself is not beneficial in the long run [29].

This false belief has been reflected by the testimonies of the participants, who they relied on the surgery, did not comply with the dietary guidelines, and did not engage in physical exercise. Surgery to lose weight may significantly increase movement and lessen the effects of osteoarthritis [30]. Individuals who are obese frequently claim having more physical activity than they actually do, as has already been shown in the literature [31]. A study found that only about 50 percent of patients committed average to intense exercise for some more than one period per week, which is lesser than the exercise of people in the overall similar demographic [32]. This is despite the fact that 89% of clients self-reported being consistently active at two years following surgery. Additionally, according to activity tracker measurements, only 11%16 of patients who have undergone MBSreally walk 10,000 steps each day, relative to 35%43 of the overall population. There are few studies on the relationship between physical exercise and weight gain, with the majority of publications citing that “reduced energy spending” was linked to obesity, despite the fact that there have been several studies on the influence of exercise on post-operative MBS results [33]. Additionally, it has been asserted that the recommended levels of exercise may be insufficient to avoid long-term weight retention following bariatric surgery. Nevertheless, it has been found that MBS patients are less physically engaged on a daily routine while having similar patterns of moderate to intense physical exercise to the general public [5].

Moreover, the participants in this study have reported lack of support from their family on top of the economic and work-related challenges, which have hindered their compliance with dietary instructions, and consequently weight regain. Recommendations for post-operative follow-up treatment with a bariatric crew have been developed by the American Society for MBS (ASMBS), which highlights the significance of follow-up management on a client’s development and results. Follow-up management assists in monitoring the patient’s development, ensuring that they listen to the advice of their medical provider, and providing tips for maintaining their weight and behavior [34]. Based on previous study, given that 50% of individuals who undergo MBS regain weight during two years following their procedure, it is not unexpected that 60% of these individuals did not obtain routine nutritional follow-up and 80% could not seek mental follow-up. In comparison, individuals who completed all of their post-surgical follow-up appointments were more effective in the longer run. In comparison to a client who was abandoned follow-up during the first year of the operation, follow-up sustained for a maximum of three years post-surgery led to an 18% greater weight reduction [35]. This finding may suggest a beneficial relationship between extended follow-up durations and enhanced health results. Following bariatric operations, the effects of support have been well researched, as shown by a number of comprehensive studies on the topic. Healthy familial, social, and medical bariatric support were found to be essential in ensuring long-term post-operative satisfaction from the client’s point of view. This provides crucial information into the viewpoints of the participants and the three main pillars thought to be essential to improve their long-term results [36]. According to these findings, a patient’s doctor and interpersonal circle, which may comprise family and friends, should urge them to attend follow-up visits and, preferably, be accompanied by those people. Encouraging the clients’ support networks to follow-up consultations could motivate them to sustain regular communication with one‘s post-operative team and also represent a reminder to clients of the favorable measures and habits addressed during discussions, which is helpful given that patients typically miss the details they have been instructed before surgery, which even farther exacerbates at one year after surgery [26].

## Conclusion and recommendations

Following MBS, weight restoration was viewed as an unanticipated and challenging event that led to feelings of frustration, embarrassment, and dissatisfaction. Our findings suggest that factors both intrinsic and extrinsic, such as economic challenges, changes in hunger, lack of support and issues with physiological and psychological health, may play a role in the deterioration of weight management. Long-term post-surgical weight control may be facilitated by interpersonal support, self-care, and lifestyle measures. The results of this qualitative investigation may serve as a hypothesis starter for subsequent quantitative research.

Despite being successful, weight regain can still happen following MBS. In order to promote long-term weight loss sustainability, the results of this paper reinforce the idea that persons who have had MBS ought to be educated of the controllable variables linked with weight rebound. The avoidance of long-term weight rebound depends on frequent, systematic, and long-term follow-up with the bariatric specialists. Inadequate dietary compliance, behavioral problems, and lack of physical activity that affect long-term weight results can all be quickly addressed with follow-up care. Future studies should establish acceptable assessments and instruments that have been verified in the bariatric community as well as a consistent concept and standard for measuring weight regain following bariatric operation. In order to give healthcare workers a better comprehension of the kinds of food items to recommend limiting and the kinds of activities to enhance, future studies should also recognize the precise foods, eating regularity, and kind of physical exercise that may be most pertinent to individuals who have had bariatric surgical procedure. Finally, psychosocial assessments should be regularly applied to enhance the involvement of the social circle in the patients’ treatment.

## Supporting information

S1 File Data availability, interview guide, and full quotations. (DOCX)
