# Supplementary material for: Understanding weight regain: Insights from Saudi patients on factors influencing post-metabolic and bariatric surgery outcomes: A qualitative study
Source: PLoS One. 2026 Feb 12;21(2):e0340120. doi: 10.1371/journal.pone.0340120 (PMC12900334; doi:10.1371/journal.pone.0340120)
Supplement: S1 File — (DOCX) [file pone.0340120.s001.docx]

**Data Availability, Interview Guide, and Full Quotations**

**Data Availability Statement**

**All relevant data—including the complete semi-structured interview guide (7 open-ended questions) and all 12 participant quotations (P1-P18)—are publicly available within the manuscript to enable full replication of the thematic analysis.**

**​**

**Interview Guide Questions**

- 1. How much did you lose weight after one year of the bariatric surgery?​
  2. How do you describe your life style after bariatric surgery?​
  3. How much do you follow a regular less fat diet after one year of the bariatric surgery?​
  4. How do you describe your dietary habits after one year of the bariatric surgery?​
  5. Do you suffer from any mental health disorder (anxiety, stress, depression) after one year of the bariatric surgery?​
  6. What are the barriers to lose weight after bariatric surgery? (Social factors, e.g., eating practices with colleagues; personal characteristics e.g., self-efficacy, motivation, knowledge; and features of the physical environment e.g., lack of availability of healthy food in onsite cafeterias, vending machines, eating behaviours).​
  7. What is the type of food intake do you follow after undergoing the bariatric surgery? (Defined per healthy eating behaviors: timing/frequency of eating, meal composition, food composition, habitual intake of energy and nutrients).​

Examples of Participant Quotations

Economic Challenges

- P3: "...you know after the whole recession started it became really hard for me to stick to a diet plan...it is really expensive to buy ingredients that you can both satisfy your cravings and stay healthy... it is way cheaper to access street food or make food at home which is usually saturated in fats..."​
- P11: "...buying proteins is really expensive these days...I love eating dairy yet low calories or non-fat dairy is completely out of my financial abilities... so I buy regular dairy products which I think it is a huge reason why I regained weight... it is the same situation with buying oats or oat bread... regular bread is much cheaper... and umm... I end up consuming eat and it leads me to crave more sugar..."​
- P7: "...I have been living off street food because I can afford it more...it saves me more money to pay for rent and gas... cooking meals costs way more than buying fast food nowadays...thats how I kind of regained the weight I guess..."​

Emotional Instability and Occupational Stress

- P12: "...It is those times when I am not in a good place mentally when I feel I want to eat and eat and eat...I submit to my cravings whether its chocolate or pizza or whatever...It makes me feel better I guess... though it is not health coping but I went through a lot after my surgery and I found my comfort in indulging unfortunately... guess I should have found a better coping style..."​
- P4: "...I have suffered with depression for the past few years...usually when I am on a low I stop eating but this time it was different... I found myself craving guess because I was deprived of my cravings for so long... it was the way that I was trying to make myself feel something... didnt help it only added to the feelings of guilt and shame..."​
- P18: "...I work night shifts...I tend to be exhausted throughout the night...I find myself snacking through the shift to pass time or to regain some energy... when I get home I would not have the power to cook so I order some fast food and eat to keep my day going..."​
- P9: "...my work is really fast paced and utterly stressful so I cant eat all day...when I get home I eat a very large meal to satisfy the hunger that I would be going through all day and I end up over eating..."​

Social Influence

- P1: "...it was really hard to look at your siblings and friends and family eating whatever they feel like eating...stuff that you really like eating and stay healthy...you can literally see your will breaking bit by bit... I could not resist.."​
- P16: "...the social gatherings and celebrations hit hard the most... whenever there is a birthday or a wedding or a holiday... everyone is happy enjoying food and I feel completely miserable for not being able to...it wasnt long till I caved in and I felt the need to give myself the freedom to eat yet I did not feel good about it every time because I was noticing the weight gain slowly..."​
- P10: "...we live in a country where gastronomy is the center of the social life...whenever I go out with my friends we are planning to eat or drink or do an activity around eating a couple meals or snacking...it wasnt good for my diet...Id either have to be antisocial or just give in to the lifestyle..."​

Lack of Support

- P14: "...my family does not understand what it takes for me to keep on track and to keep my mind and body balanced through all of this...when I decided to go for the surgery I was really struggling to convince my family how important this is for me and after that they wont understand that they need to be involved in my journey to support me emotionally and actually through actions around the house...like give me options"​
- P2: "...I mean my family has to kind of diet with me when I am around...like cooking stuff that can actually fit my diet... encourage me to stay healthy rather than just do whatever they want and when I ask them for help theyre like this is your choice not ours..."​
- P17: "...it is really hard to feel like you are on your own...you cave...my father especially is caught up in his ways and is not willing to bend some rules for my sake...so I am obliged to eat whatever theyre eating and it is fatty and full of carbs...when I ask for healthy food he would get to sarcastic and mock me... and I rely on them for food its not like I have any other choice..."​

False Beliefs and Decreased Physical Activity

- P8: "...I cant believe I was so unrealistic in how I expected that the surgery alone would do the trick and I just felt like why would I have to exercise...instead I was just stick to my regular routine during the day and I guess that was not very good..."​
- P15: "...the doctor told me that I would have to exercise...but I did not...when I first decided to go for the surgery I went in with the toxic optimism that it is the miraculous solution to my weight and that it is like a magic wand that will strip off all those kilos on its own...so I did not exercise and I ended up actually regaining all the weight that I would have potentially lost if I was more careful and keen..."​

This represents everything shareable; other sections contain sensitive details like full demographics or potential raw data not suitable for disclosure.
